# Effects of Endocrine-Disrupting Heavy Metals on Human Health

**DOI:** 10.3390/toxics11040322

**Published:** 2023-03-29

**Authors:** Dongling Liu, Qianhan Shi, Cuiqing Liu, Qinghua Sun, Xiang Zeng

**Affiliations:** 1 School of Basic Medical Science, Zhejiang Chinese Medical University, 548 Binwen Road, Hangzhou 310053, China; liudongling8511@126.com; 2 School of Public Health, Zhejiang Chinese Medical University, 548 Binwen Road, Hangzhou 310053, China; 202211115611007@zcmu.edu.cn (Q.S.); liucuiqing@zcmu.edu.cn (C.L.); qhsun@zcmu.edu.cn (Q.S.)

**Keywords:** endocrine disruption, heavy metals, elements, thyroid hormone, estrogen, health

## Abstract

Heavy metals play an important endocrine-disrupting role in the health consequences. However, the endocrine-disrupting mechanism of heavy metals is unclear. There are long-term and low-level metal/element exposure scenes for the human body in real life. Therefore, animal models exposed to high doses of heavy metals may not provide key information to elucidate the underlying pathogeny of human diseases. This review collects current knowledge regarding the endocrine-disrupting roles of heavy metals such as lead (Pb), cadmium (Cd), arsenic (As), mercury (Hg), nickel (Ni), copper (Cu), zinc (Zn), and manganese (Mn), summarizes the possible molecular mechanisms of these endocrine-disrupting chemicals (EDCs), and briefly evaluates their endocrine toxicity on animals and humans.

## 1. Introduction

Heavy metals, defined as elements of a density greater than 5 g/cm^3^, are a group of metals and metalloids that are universally used in industrial production and consumer goods. They are considered one of the major environmental pollutants due to the current limited level of industrial manufacturing, the low recycling rate, informal dismantling activities and the inadequate and unbalanced regional socio-economic conditions. In other words, heavy metals can accumulate in environmental media such as air, dust, soil, water, and sediment for a long time [1]. Notably, unlike organic contaminants, heavy metals have non-biodegradable characteristics in the natural environment, which means they usually cannot be converted into less dangerous end products. In addition, they can be enriched thousands of times through biological amplification of the food chain and eventually enter the human body through inhalation, ingestion, and dermal absorption. Although some heavy metals such as Zn, Cu, and Mn are microelements needed for normal life activities and homeostasis, most of them, such as Pb, Cd, and Hg, are poisonous to humans even at very low doses [2]. It is worth mentioning that all heavy metals are toxic to the human body when their concentrations in the body exceed a certain concentration or above a particular threshold. Moreover, the accumulation of heavy metals will interact and impact the activity of enzymes, proteins, and metabolism, subsequently causing biochemical, morphological, and functional changes [3]. Furthermore, different heavy metals tend to accumulate in various tissues and organs in the body, and eventually leading to chronic poisoning when it increases to a certain concentration. Long-term exposure to heavy metals will stimulate the body to produce oxidative stress and inflammatory reactions, which are associated with a variety of health consequences, such as neurotoxicity and respiratory, cardiovascular, reproductive, and renal toxicity [4,5,6]. However, to date, little is known about the endocrine disruption effect of heavy metals on organisms.

**Figure 1:**
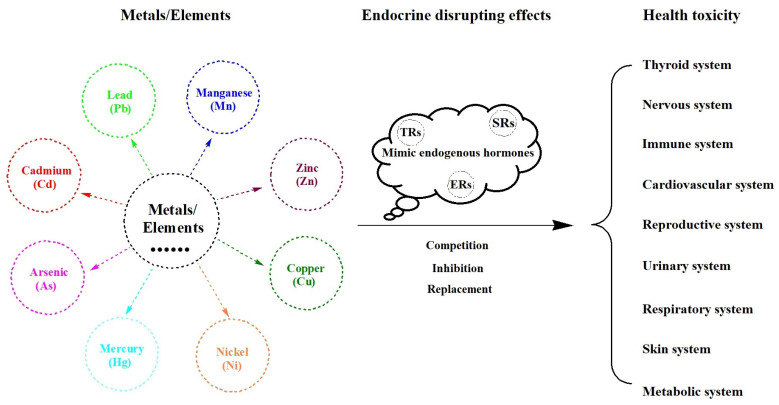
Endocrine-disrupting effects of heavy metals on health.

Endocrine-disrupting chemicals (EDCs) are exogenous substances that can mimic endogenous hormones with similar structures or activities to influence their synthesis and metabolism. They can interact with hormone receptors, such as thyroid hormone receptors (TRs) and sex hormone receptors (SRs), mainly including estrogen receptors (ERs), androgen receptors (ARs), and progestational receptors (PRs), alter the functions of the endocrine system, and generate adverse health effects by synergizing or antagonizing endocrine hormonal effects (Figure 1) [7,8,9]. They have the ability to act as ligands and attach to specific hormone receptors, which bind to response elements in target genes, producing undesirable downstream effects by regulating gene expression. Additionally, hormones are synthesized and secreted by a variety of glands of the endocrine system. The maintenance of hormone homeostasis cannot be achieved if these exogenous EDCs interfere with the generation, transport, or metabolism of native hormones in the human body. Moreover, EDCs are ubiquitous substances that are found in our daily items, including heavy metals, pesticides, plasticizers, pharmaceuticals, personal care products, food products, and food packaging. The endocrine system is well known to play an important role in regulating metabolic processes, adjusting extracellular fluid (amount and composition), dominating growth and development, altering reproductive function, and maintaining endocrine homeostasis (resistance and adaptability). In other words, EDCs are closely related to our health status, especially in the case of over-dose exposure when the human body receives too many EDCs to metabolize or eliminate. Of these, heavy metals have a major impact on endocrine health [10,11,12]. Therefore, the potential endocrine interference mechanism of heavy metals needs to be revealed to reduce or eliminate its adverse effects.

Evidence has been documented that heavy metals can serve as endocrine disruptors in humans and animals, affecting hormone homeostasis and causing endocrine imbalance [13,14,15]. The best-characterized heavy metals as endocrine disruptors include Pb, Cd, As, Hg, Cu, Ni, Zn, and Mn. It is well known that the thyroid is the largest endocrine gland with a substantial blood supply, which is crucial for the regulation of human growth and metabolism. Meanwhile, thyroid hormones are the endpoint most frequently evaluated for heavy metals, followed by the nervous, immune, cardiovascular, urinary, digestive, and reproductive systems. There are inconsistent results between different heavy metals and even in the same elements. Therefore, a dose-response analysis of heavy metals is needed to obtain a more accurate conclusion. This review highlights the current state of knowledge regarding the role of heavy metals in human endocrine health, which may shed light on the mechanisms of endocrine toxicity of heavy metals in organisms.

## 2. Health Effects of Heavy Metals

Although exposure to heavy metals with endocrine-disrupting effects is present across the lifespan, the sensitive window period of each element has a different and unique vulnerability. Exposure to heavy metals in early life has particularly adverse effects on health due to its characteristics of accumulation and irreversibility. The exposure dose and the combined exposure are also key factors to be considered when paying attention to the health effects of heavy metals.

### 2.1. Lead (Pb)

As a traditional and typical heavy metal, Pb is ubiquitous in gasoline, batteries, glass, paints, coatings, pesticides, and plumbing fixtures. Although many efforts, such as the prohibition of leaded gasoline and paints, have been made to reduce environmental Pb levels, Pb is still abundant in environmental media and organisms. Additionally, some academic institutions indicated that 5 μg/dL of blood Pb level is considered acceptable [16]. However, some research data have suggested that there is no safe level of Pb in the human body [17]. It is worth mentioning that inorganic lead and organic lead are Group 2A (suspected human carcinogen) and Group 3 (not classifiable as a human carcinogen) carcinogens according to the IARC of WHO agent classification list, respectively [18]. Moreover, Pb, an endocrine disruptor, is a non-degradable toxic substance in the environment, and its health damage is usually irreversible [19,20,21]. Therefore, the endocrine toxicity of Pb still exists and needs more attention and investigation. Previous studies have primarily investigated the impact of Pb on the endocrine (thyroid hormones and sex hormones), nervous (neurotransmitters/neuropeptides), and immune systems (inflammatory cytokines) [22,23,24,25,26]. Specifically, there are inconsistent epidemiological results in the association between Pb and thyroid hormones [27,28,29]. Exposure to Pb is positively associated with sex hormones such as testosterone and sex-hormone-binding globulin [24]. In addition, exposure to Pb is significantly associated with alterations in IQ, neurotransmitters, and cognitive and behavioral scores in children [29,30,31,32]. Moreover, exposure to Pb markedly alters inflammatory cytokine levels in children [33,34,35,36]. Note that adult patients with Pb poison may experience changes in their thyroid function, including a decrease in thyroid-stimulating hormone (TSH) and an increase in thyroxine (T4) [37]. Similarly, workers have significantly higher levels of Pb in their blood than the control group and are at risk for hyperthyroidism [38]. Taken together, Pb can cause damage to multiple human systems, of which the endocrine system is particularly impacted.

### 2.2. Cadmium (Cd)

Cd naturally exists as a cadmium sulfide ore, followed by Zn, Pb, and Cu ores, and is used mainly for electroplating of steel, iron, copper, brass, and other heavy metals, then batteries, pigments, plastic stabilizers, coatings, pesticides, and phosphate fertilizers. Environmental pollution, tobacco smoking, and diet are the main sources of Cd exposure in the general population. It can be absorbed into the blood, which mainly combines with red blood cells, and further distributed to other organs through the circulatory system. The top organs that store Cd in the body are the liver, kidney, lung, skeleton, and thyroid gland, and the Cd present in these organs makes up over 60% of the total cadmium in the body. It is worth mentioning that the half-life of Cd is approximately 15–30 years [39]. Like Pb, Cd is a metal that humans do not require for normal physiological activities of the body and can accumulate in the human body via the food chain and drinking water [20]. In other words, exposure to Cd is harmful to human health. Moreover, Cd and its compounds are carcinogens assigned to Group 1 (human carcinogen), according to the International Agency for Research on Cancer (IARC) of WHO [18,40,41]. Furthermore, Cd prefers to deposit in the thyroid gland and has both endocrine-disrupting effects and thyroid toxicity because of its high binding affinity to cysteine-rich proteins and metallothionein in the thyroid gland [42,43]. Long-term exposure to Cd can lead to thyrotoxicity [44]. Previous studies indicated that the reference value of urinary Cd in humans is not higher than 1 μg/g (European Food Safety Authority) or 2 μg/g creatinine [the National Health and Nutrition Examination Survey (NHANES) database] or 5.24 μg/g (WHO) [45,46]. A recent study suggested that Cd exposure promotes thyroid follicular cell pyroptosis by inhibiting Nrf2/Keap1 signaling and, therefore, may alter the structure of thyroid tissue and endocrine function [47]. Along with other heavy metals, Cd should be considered an estrogen disruptor that mimics the actions or biological functions of estrogen hormones with estrogenic activities. For example, Cd^2+^ and Co^2+^ can substitute for Zn^2+^ in the zinc finger DNA-binding domain of the ERs to associate with the estrogen response [48]. Unlike animal experiments, there are few human studies on Cd. For example, Nie et al. found that, in women, blood Cd levels were linked with hypothyroid status and thyroglobulin antibodies, while blood Pb concentrations were positively associated with TSH and thyroid peroxidase antibodies [49].

### 2.3. Arsenic (As)

As being a poisonous metalloid that is naturally and widely distributed in the environment, inorganic arsenic is a ubiquitous environmental EDC with a globally high exposure level in drinking water that exceeds the reference of 10 μg/L settled by the WHO, which has attracted worldwide health concern [50]. It ranks first on the priority list of substances issued by the agency for toxic substance and disease registry due to its high toxicity [51]. Additionally, it is a well-known toxin and carcinogen that belongs to Group 1 (human carcinogen), which can enter the human body through several routes, such as drinking water, skin contact, breathing air, and dietary food [18,52]. Endocrine disruption may be a potential mechanism that mediates the link between exposure to As and health outcomes such as skin, bladder, lung, liver, kidney, prostate, and other cancers, specifically through the interaction of As with TRs and ERs and subsequent activation of thyroid or estrogen-regulated genes [53,54,55]. For example, Zhang et al. and Guo et al. demonstrated that chronic exposure to As interferes with several estrogen levels, such as estradiol (E2), progesterone (P), luteinizing hormone (LH), follicular estrogen (FSH), gonadotropin-releasing hormone (GnRH), pituitary prolactin (PRL), and cortisol (Cort) in female rats [56,57]. In addition, methylated metabolites of inorganic arsenic not only mediate the activation of the glucocorticoid receptor but also reduce nuclear translocation and DNA binding [58]. Along with endocrine disruption, other pathways such as oxidative stress, inflammatory response, DNA impairment, and cell signaling and proliferation also play an important role in the pathogenesis of diseases when exposed to As [54]. Interesting, like a coin has two sides, As is a double-edged sword. For instance, arsenic trioxide (As_2_O_3_) can act as a demethylation agent that inhibits the expression of ERα and DNA-methyl-transferase-1, and reduces the volumes of xenograft tumors in As_2_O_3_-treated mice compared to those of the untreated mice [59].

### 2.4. Mercury (Hg)

Hg is a highly toxic and non-essential element that can be found all over the world in a wide range of environmental media as well as in the food chain (particularly fish). It naturally exists in three patterns of Hg elements (metallic), inorganic Hg (salt), and organic Hg. It is worth mentioning that Hg is very little distributed in nature and rarely exists in the pure metal state, mostly in the form of compounds. Common Hg-containing minerals are mercury sulfide (known as cinnabar), chlorosulfuric mercury ore, and thiosulfuric antimony mercury ore. As a naturally occurring metal, Hg is stored in environmental media such as air, water, and soil and accumulates in humans via the food chain. The Hg concentration of 1.0 mg/kg in human hair is the threshold recommended by the WHO [60]. In particular, Hg and inorganic Hg (Group 3 carcinogens, not classifiable as human carcinogens) can be converted to methylmercury [MeHg (Group 2B carcinogens, possible human carcinogens)] with stronger toxicity under certain conditions [18]. For example, inorganic Hg in water and sediment can synthesize MeHg under the action of microorganisms [61]. The emerging evidence for Hg-induced toxicity is mainly neurotoxicity, reproductive toxicity, histone modification, siRNA and DNA damage, and methylation [62,63,64,65,66]. In addition, prenatal and postnatal exposure to Hg is significantly associated with testosterone levels and Tanner stage > 1 in children [67]. High-dose Hg-induced disruption of thyroid function disruption is achieved via damage to the structure of the thyroid and alteration of expression of relevant genes in Chinese toads [68]. HgCl_2_ exposure leads to a decrease in sperm quality, pregnancy rate, mean litter size, and survival rate in male mice [69]. Total Hg concentrations in red blood cells were positively linked with thyroxine (T4) and inversely related to triiodothyronine (T3) in the western pond turtle, which is consistent with Hg-induced disruption of T4 deiodination [70]. Collectively, further studies are needed to prove the endocrine-disrupting effects of Hg on health, which may shed light on potential mechanisms and therapeutic targets of Hg toxicity.

### 2.5. Other Heavy Metals

In addition to the elements mentioned above, nickel (Ni), copper (Cu), zinc (Zn), and manganese (Mn) have also received long-term attention from the public and academia [71,72,73,74,75]. For example, Ni was significantly associated with the incidence of all breast cancers [76]; Exposure to Cu can lead to endocrine, reproductive disruption in zebrafish [77]; Mn triggered an endocrine disruption response manifested by a decrease in plasma concentration of 17α-OHP and cortisol [78]. The molecular mechanism of the endocrine-disrupting effect of heavy metals/elements is not yet clear. The health burden caused by heavy metals is realistic, difficult, and unavoidable. The hormonal disrupting effects of heavy metals have a serious impact on multiple systems and organizations, which cannot be ignored to maintain health.

## 3. Conclusions

The endocrine-disrupting effects of heavy metals have been documented based on the results in epidemiology and experiments in vivo and in vitro. Most animal studies are mainly focused on a single heavy metal, which is far from real-life exposure to combined metal exposure. Only a few epidemiological studies reported the endocrine-disrupting effects of each heavy metal. This knowledge may improve further advances in investigating the endocrine-disrupting mechanism of heavy metals. The potential endocrine-disrupting mechanism of heavy metals is still unclear, and more epidemiological studies are needed to confirm the existing findings and current hypothesis.

## Data Availability

Not applicable.
